# Exploring novel targets of sitagliptin for type 2 diabetes mellitus: Network pharmacology, molecular docking, molecular dynamics simulation, and SPR approaches

**DOI:** 10.3389/fendo.2022.1096655

**Published:** 2023-01-09

**Authors:** Jian-hong Qi, Pu-yu Chen, Ding-yuan Cai, Yi Wang, Yue-lei Wei, Su-ping He, Wei Zhou

**Affiliations:** Department of Pharmaceutics, China Pharmaceutical University, Nanjing, China

**Keywords:** ACE2, sitagliptin, type 2 diabetes mellitus, network pharmacology, molecular docking, molecular dynamics simulation, SPR

## Abstract

**Background:**

Diabetes has become a serious global public health problem. With the increasing prevalence of type 2 diabetes mellitus (T2DM), the incidence of complications of T2DM is also on the rise. Sitagliptin, as a targeted drug of DPP4, has good therapeutic effect for T2DM. It is well known that sitagliptin can specifically inhibit the activity of DPP4 to promote insulin secretion, inhibit islet β cell apoptosis and reduce blood glucose levels, while other pharmacological mechanisms are still unclear, such as improving insulin resistance, anti-inflammatory, anti-oxidative stress, and anti-fibrosis. The aim of this study was to explore novel targets and potential signaling pathways of sitagliptin for T2DM.

**Methods:**

Firstly, network pharmacology was applied to find the novel target most closely related to DPP4. Semi-flexible molecular docking was performed to confirm the binding ability between sitagliptin and the novel target, and molecular dynamics simulation (MD) was carried to verify the stability of the complex formed by sitagliptin and the novel target. Furthermore, surface-plasmon resonance (SPR) was used to explored the affinity and kinetic characteristics of sitagliptin with the novel target. Finally, the molecular mechanism of sitagliptin for T2DM was predicted by the enrichment analysis of GO function and KEGG pathway.

**Results:**

In this study, we found the cell surface receptor―angiotensin-converting enzyme 2 (ACE2) most closely related to DPP4. Then, we confirmed that sitagliptin had strong binding ability with ACE2 from a static perspective, and the stability of sitagliptin―ACE2 complex had better stability and longer binding time than BAR708―ACE2 in simulated aqueous solution within 50 ns. Significantly, we have demonstrated a strong affinity between sitagliptin and ACE2 on SPR biosensor, and their kinetic characteristics were “fast binding/fast dissociation”. The guiding significance of clinical administration: low dose can reach saturation, but repeated administration was needed. Finally, there was certain relationship between COVID-19 and T2DM, and ACE2/Ang-(1-7)/Mas receptor (MasR) axis may be the important pathway of sitagliptin targeting ACE2 for T2DM.

**Conclusion:**

This study used different methods to prove that ACE2 may be another novel target of sitagliptin for T2DM, which extended the application of ACE2 in improving diabetes mellitus.

## 1 Introduction

Diabetes is a metabolic disease characterized by chronic hyperglycemia caused by multiple causes, which can lead to cardiovascular and cerebrovascular diseases, diabetic nephropathy, diabetic retinopathy and other chronic complications (1). Diabetes has become a major global public health problem, seriously affecting people’s daily life. In the world, the prevalence of diabetes is increasing year by year, and the number of patients with type 2 diabetes mellitus (T2DM) accounts for more than 90% of diabetic patients (2, 3). At present, the drugs for T2DM are divided into two categories: traditional drugs (e.g., sulfonylureas, thiazolidinediones, biguanides, and insulin) and new target drugs (e.g., multiple incretin agonists, glucokinase agonists, and glucagon receptor antagonists) (4).

Sitagliptin was approved by the Food and Drug Administration (FDA) in 2006 and can be used alone or in combination with other drugs to treat T2DM and improve blood glucose control (5). It was the first dipeptidyl peptidase IV (DPP4) inhibitor for the treatment of T2DM, which mainly reduced the degradation of glucagon-like peptide-1 (GLP-1) by selectively inhibiting the activity of DPP4, thereby exerting hypoglycemic effect (6). DPP4, also known as CD26, was a 110 kDa type II transmembrane glycoprotein expressed in various tissues (e.g., brain, endothelium, heart, intestine, kidney, liver, lung, skeletal muscle, pancreas, placenta, and lymphocytes) (7). GLP-1 was an endogenous physiological substrate of DPP4. GLP-1 mainly stimulated insulin secretion and inhibited glucagon secretion, thereby limiting postprandial blood glucose fluctuations. As a physiological regulator of appetite and food intake, it also inhibited gastrointestinal peristalsis and gastric acid secretion. GLP-1 secretion increased rapidly after meals, but about 75% of the secreted peptides were degraded by DPP4 on the lumen surface of endothelial cells, and only 25% reached the portal circulation. 50% of the remaining GLP-1 was further degraded under the action of liver DPP4 and soluble DPP4 (8). Therefore, DPP4 was closely related to T2DM, and we believed that finding DPP4-related targets may provide a new idea for the development of T2DM targeted drugs.

In this study, we found a novel target of sitagliptin for T2DM closely related to DPP4 from multiple databases, and explored the affinity and kinetics characteristics of sitagliptin and the novel target by molecular docking, molecular dynamics simulation (MD) and surface-plasmon resonance (SPR) approach, and predicted the molecular mechanism of sitagliptin in the treatment of T2DM by network pharmacology, which provided more feasible drug targets for T2DM.

## 2 Materials and methods

### 2.1 Discovery of potential targets

With “sitagliptin” as the keyword, the ingredient-targets were predicted in PharmMapper (http://www.lilab-ecust.cn/pharmmapper/) and Super-PRED (https://prediction.charite.de/) databases (9, 10); using the keywords of “type 2 diabetes mellitus” and “type 2 diabetes”, the known disease-targets were searched in DrugBank (https://go.drugbank.com/) and TTD (http://db.idrblab.net/ttd/) databases (11, 12). Then, jvenn (http://www.bioinformatics.com.cn/static/others/jvenn/) online platform was used to extract intersection targets of ingredient-targets and disease-targets (13). Finally, the potential targets for sitagliptin in the treatment of T2DM were obtained, and the symbol names were standardized in UniProt (https://www.uniprot.org/) database (14).

### 2.2 Construction of relationship network for potential targets

To obtain DPP4-related targets, we constructed a protein-protein interaction (PPI) network for potential targets, and explored their relationship. The potential targets were imported into STRING (https://cn.string-db.org/) online platform, and the following conditions were defined: organism (homo sapiens), medium confidence (0.400), and the results were visualized by Cytoscape software (15). Finally, we found the novel target closely related to DPP4.

### 2.3 Molecular docking

Molecular docking originates from the “lock-key” model, which is a computer simulation method to analyze the interaction between targets and compounds through the 3D crystal structure of targets. From a static perspective, we used AutoDock Vina software and python script to explore the binding ability and binding mode between sitagliptin and the novel target (16). Finally, we obtained the complex structure of ligand compound and receptor protein.

### 2.4 Molecular dynamics simulation

MD can simulate the molecular trajectory in different real environments, and obtain the information such as free binding energy, system stability, bonding type and amino acid residue flexibility. In simulated body water environment, we used Gromacs software and program packages to explore the stability of the complex structure formed by sitagliptin and the novel target, thus supporting the results of molecular docking (17).

### 2.5 SPR assay analysis

To verify the affinity and kinetics between sitagliptin and the novel target, we performed this experiment using Biacore T200 (GE Healthcare) instrument based on SPR, and used GraphPad Prism 8.0 software to visualize the data results (18, 19).

Firstly, the amino acid sequence of the target protein was searched by Uniprot database, and the sequence was imported into Expasy (https://web.expasy.org/) website to calculate the isoelectric point of the protein (20). Then, the preconcentration experiment was carried out to determine the optimal coupling conditions. The target protein was coupled to CM5 chip (GE Healthcare) using the Immobilization module in Biacore T200 Control Software, and the reference channel was set to deduct the background. The chip was activated by EDC/NHS (GE Healthcare), and the uncoupling site was blocked by ethanolamine (GE Healthcare).

Sitagliptin (HPLC ≥ 98%, Shanghai yuanye Bio-Technology Co., Ltd) was dissolved in DMSO (VWR) solution and diluted with PBS-P+ (GE Healthcare) solution to the desired compound concentration (1×PBS-P+, 5% DMSO). The affinity and kinetics of sitagliptin with the novel target protein were tested by LMW kinetics module in Biacore T200 Control Software, and extra wash after injection with 50% DMSO was added.

### 2.6 Enrichment analysis of gene ontology (GO) function and Kyoto Encyclopedia of Genes and Genomes (KEGG) pathway

The potential targets for sitagliptin in the treatment of T2DM were imported into Metascape (http://metascape.org/) online platform for enrichment analysis, and the following parameters were defined: species (H. sapiens), min overlap (3), p value cutoff (0.1), min enrichment (1.5), and then the results were visualized by bioinformatics (http://www.bioinformatics.com.cn/) online platform (21).

### 2.7 Construction of drug-targets-signaling pathways network

In order to reflect the “holistic view” of the molecular mechanism for sitagliptin in the treatment of T2DM, we imported sitagliptin, potential targets and signaling pathways into Cytoscape software to construct the drug-targets-signaling pathways network. In addition, we further explored the novel target-related signaling pathways to provide reference for mechanism research.

## 3 Results

### 3.1 Discovery of potential targets

154 ingredient-targets (norm fit > 0.5) were predicted and screened from PharmMapper database, and 148 ingredient-targets (probability > 50%) were predicted and screened from Super-PRED database. All the symbol names were gathered, 15 duplicate values were excluded, and 287 unique targets for sitagliptin were finally obtained.

100 known disease-targets were obtained from DrugBank database, and 85 known disease-targets were obtained from TTD database. All symbol names were gathered, and 17 duplicate values were excluded. Finally, 168 unique targets for T2DM were obtained (each target had corresponding drugs).

**Figure 1:**
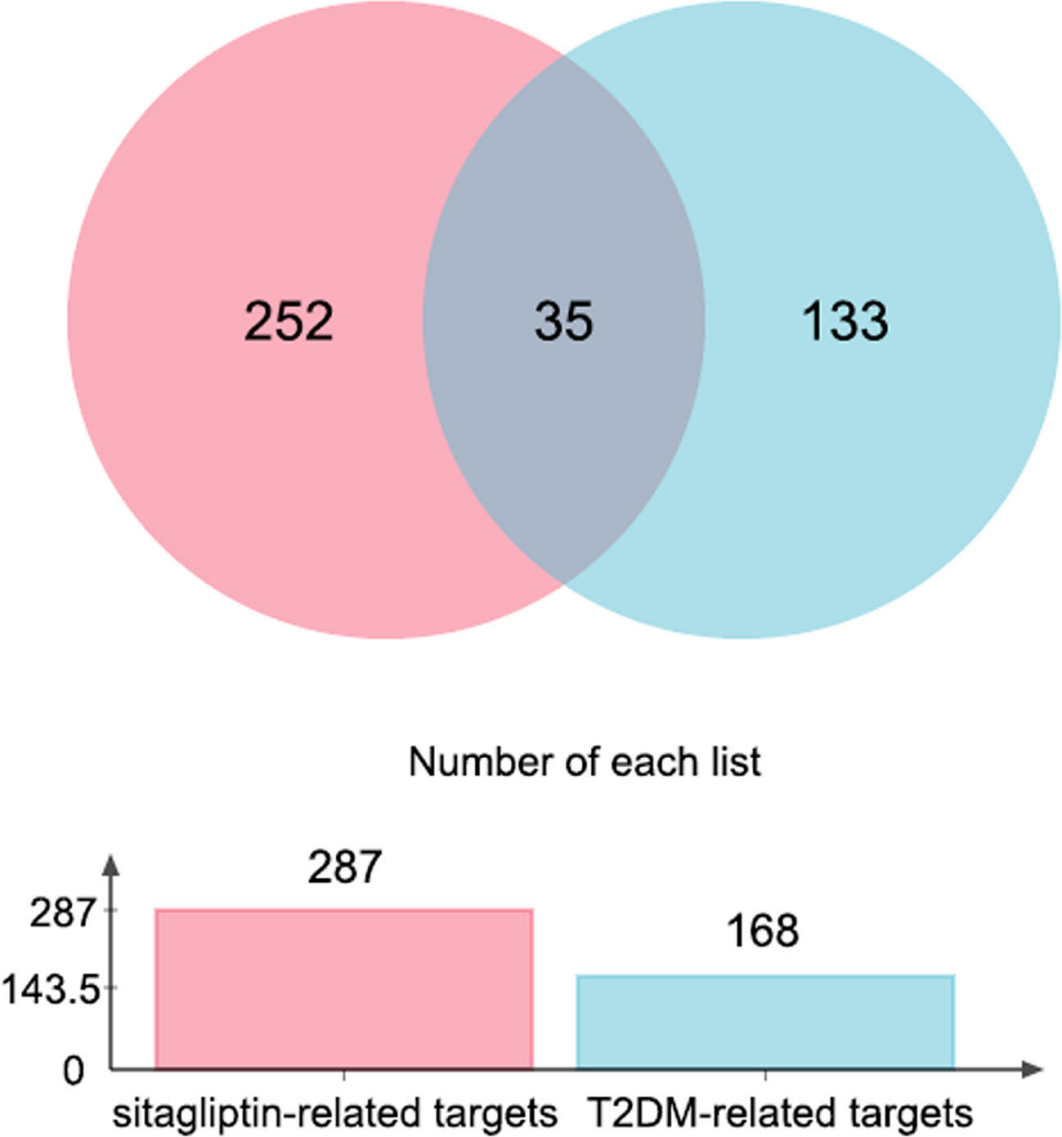
Venn graph of sitagliptin-related targets and T2DM-related targets.

Through jvenn online platform, 35 intersection targets of ingredient-targets and disease-targets were obtained, which may be potential targets for sitagliptin in the treatment of T2DM (**Figure 1**).

### 3.2 Construction of relationship network for potential targets

**Figure 2:**
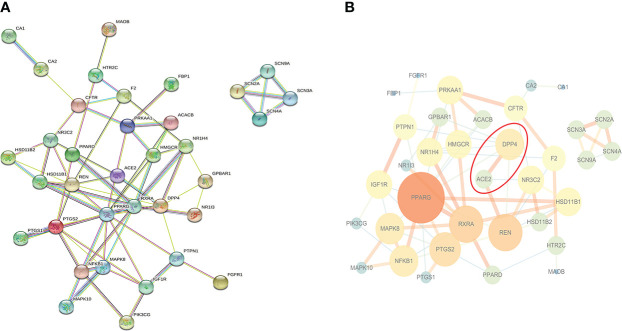
PPI network of potential targets. **(A)** Original graph from STRING online platform: Nodes are proteins, and edges of different colors are the interaction between proteins; **(B)** Relationship network graph processed by Cytoscape software: The larger the node and the darker the color, the more important the node is; the thicker the edge and the darker the color, the closer the relationship between nodes.

35 potential targets were imported into STRING online platform to obtain tsv. format file and visualized by Cytoscape software (**Figure 2**). Through the network topology analysis, it was found that the network contains 35 nodes and 70 edges. The average of AverageShortestPathLength was 2.47, the average of BetweennessCentrality was 0.05, the average of ClosenessCentrality was 0.46, and the average of Degree was 4. Importantly, it can be seen from **Figure 2B** that angiotensin-converting enzyme 2 (ACE2) was most closely related to DPP4, and they were co-expressed, which may have similar biological functions. Therefore, it was speculated that ACE2 may also be a novel target for sitagliptin in the treatment of T2DM.

### 3.3 Static molecular modeling of BAR708―ACE2 and sitagliptin―ACE2

Based on apo ACE2, we constructed a receptor model for ACE2 agonist with reference to the structure of BAR708―ACE2 (22). Here, BAR708 was used as a positive control to verify the binding ability and binding mode between sitagliptin and ACE2 from a static perspective.

Firstly, the crystal structure of ACE2 (PDB ID: 1R42) was downloaded from the RCSB PDB (https://www.rcsb.org/) database (23, 24). We downloaded the molecular structure of sitagliptin from PubChem database (https://pubchem.ncbi.nlm.nih.gov/) and converted it to a 3D structure file using OpenBabel software. The structure of ACE2 was processed by PyMOL software as follows: removing water, Cl^-^, and Zn^2+^, deleting irrelevant component structures (UNK, NAG), and extracting the original ligand BAR708 from BAR708―ACE2. Then, we used AutoDockTools to process ACE2, BAR708, and sitagliptin, respectively. ACE2: adding hydrogen atoms, computing charges, assigning AD4 atom type, finding and merging nonpolar hydrogens, repairing amino acid residues, and saving them as PDBQT structural files. BAR708 and sitagliptin: adding hydrogen atoms, computing charges, setting rotatable chemical bonds, minimizing energy, and saving them as PDBQT structural files. The relevant parameters of the active pocket for molecular docking were determined with BAR708 as the center. The center coordinates were (72.554, 62.452, 41.772), the exhaustiveness of the program was 8, the number of modes was 9, and the maximum energy difference was 3. Here, we used AutoDock Vina software and python script to realize the molecular docking process.

**Figure 3:**
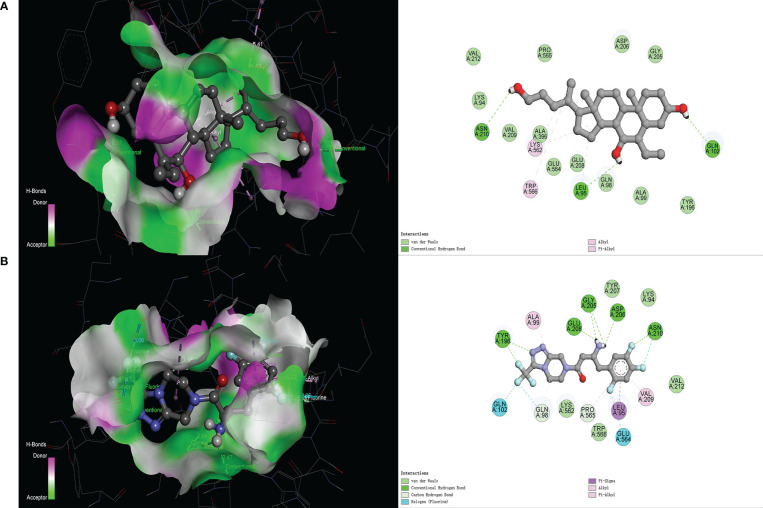
Molecular docking of different ligands with ACE2. **(A)** BAR708―ACE2, **(B)** Sitagliptin―ACE2. The 3D graphs show the distribution of hydrogen donors and acceptors in the active pocket of ACE2, and the 2D graphs show the amino acid residues and types of bonding in the active pocket.

In the molecular docking score, we obtained the best conformation of sitagliptin, and the score −8.7 kcal/mol was significantly better than −8.1 kcal/mol (BAR708). It is generally believed that the score is lower than −5 kcal/mol, indicating that a receptor has good binding ability with a ligand (25). As shown in **Figure 3**, the 3D structure of the results was displayed by PyMOL software, and the 2D structure was displayed by Discovery Studio 4.5 software. From the above figures, sitagliptin can be well embedded in the active pocket of ACE2, and the bonding types were mainly hydrogen bond, halogen bond and hydrophobic interaction. As an important index to measure the binding ability of proteins to small molecules, hydrogen bonds were included in the scope of this study. Among them, BAR708 formed hydrogen bonds with three amino acid residues (ASN210, LEU95 and GLN102), and sitagliptin formed hydrogen bonds with five amino acid residues (ASN210, ASP206, GLY205, GLU208 and TYR196). In short, sitagliptin had strong binding ability with ACE2.

### 3.4 Dynamic molecular modelling of BAR708―ACE2 and sitagliptin―ACE2

To explore the stability of the complex structures formed by sitagliptin and ACE2, MD were performed using Gromacs software and program packages.

We used PRODRG server (http://davapc1.bioch.dundee.ac.uk/cgi-bin/prodrg) and gmx pdb2gmx program to generate ligand and receptor topology files respectively, and selected GROMOS96 43a1 force field to construct receptor-ligand complexes topology files (26). The gmx editconf and gmx solvate programs were used to construct dodecahedral boxes and SPC water models, respectively (26). Based on the gmx genion program, ions were added to ensure the electroneutrality of system. The steepest descent minimization method was performed to minimize the energy of system. The V-rescale coupling method was chosen to realize the balance of heat bath (NVT, Number of particles, Volume, and Temperature), and the following parameters were defined: the reference temperature was 300 K, the step length was 2 fs, and the running time was 100 ps. The Parrinello-Rahman coupling method was chosen to realize the balance of pressure bath (NPT, Number of particles, Pressure, and Temperature), and the running time was 100 ps (27). Particle mesh Ewald (PME) algorithm was used to calculate the long-range electrostatic interaction; the cut-off algorithm was used to calculate the short-range electrostatic interaction, and the cut-off radius was 1.2 nm (28). Starting from the balanced result file of NPT, the MD time was set to 50 ns, and the trajectory file of the simulation operation was saved. The gmx grompp and mdrun programs were used to simulate the molecular dynamics of complex solution systems.

**Figure 4:**
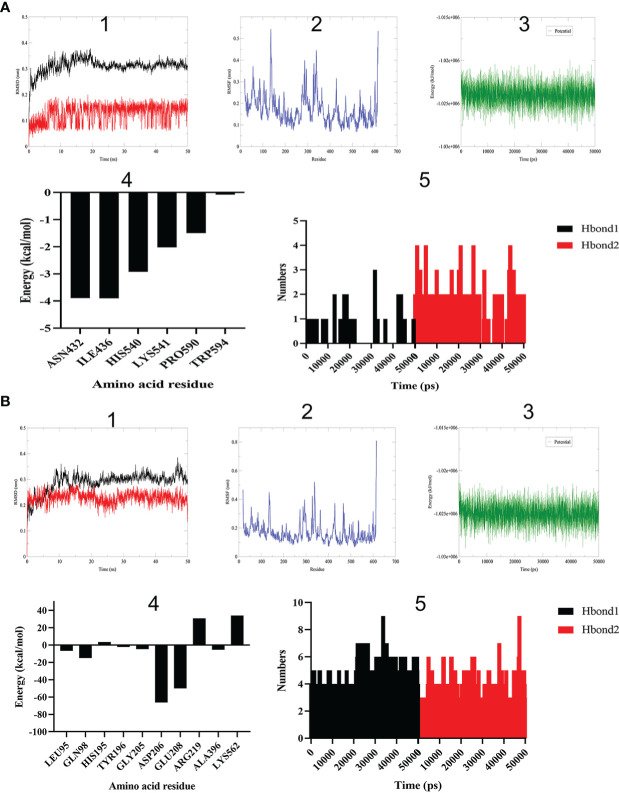
Molecular dynamics simulation of different ligands with ACE2. **(A)** BAR708―ACE2. **(B)** Sitagliptin―ACE2. (1) RMSD reveals the position change between real-time conformation and initial conformation of protein-ligand complex (black: complex; red: ligand). (2) RMSF shows the fluctuation amplitude of each atom relative to the average position. (3) Energy change of system in SPC water model. (4) Amino acid residue energy decomposition of protein complexes formed by sitagliptin and ACE2. (5) Number variation of hydrogen bonds during simulation time (Hbond1: hydrogen donor-acceptor distance less than 0.35 nm and angle less than 30 degrees. Hbond2: except for Hbond1, hydrogen donor-acceptor distance less than 0.35 nm).

Firstly, root mean square deviation (RMSD) can reveal the position change between real-time conformation and initial conformation of protein-ligand complex during MD simulation. The variation trend of RMSD for protein-ligand complex was an important characterization for judging the stability of MD simulation. As shown in **Figure 4A**, the backbone RMSD of BAR708―ACE2 complex reached the maximum value of 0.38 nm at 19 ns, then stabilized around 0.31 nm, and the RMSD amplitude was less than 0.2 nm; the RMSD value of BAR708 reached the maximum value of 0.20 nm at 19 ns, although the RMSD value of BAR708 had large amplitude, it was still within 0.2 nm. As shown in **Figure 4B**, the backbone RMSD value of sitagliptin―ACE2 complex reached a stable state after 11 ns and was near 0.30 nm. During the MD time, the RMSD value of sitagliptin quickly reached a stable state after 5 ns, and was near 0.23 nm. Compared with BAR708―ACE2 and sitagliptin―ACE2, the stability of the complex formed by sitagliptin and ACE2 was the best, and the RMSD of the complex was close to that of the ligand, indicating that sitagliptin did not interfere with the stability of the complex. Root mean square fluctuation (RMSF) showed the fluctuation amplitude of each atom relative to the average position. Here, RMSF was used to measure the flexibility of amino acid residues of receptor protein, and characterize the flexibility and motion intensity of amino acid residues in the whole simulation process. The larger the RMSF value, the more obvious the atomic free movement, indicating that the flexibility of amino acid residues was greater. Except for the N-terminus and C-terminus, the amino acid residues of BAR708―ACE2 and sitagliptin―ACE2 showed similar flexibility and motion intensity, so sitagliptin may activate the amino acid residues of ACE2 at active sites. The potential energy of SPC water model system constructed by sitagliptin―ACE2 was approximately equal to that of BAR708―ACE2, and was stable around −1.03e+006 kJ/mol. In addition, we extracted the conformation of the last frame of the sitagliptin―ACE2 and BAR708―ACE2 to explore the energy decomposition of amino acid residues, respectively. The former showed better binding free energy distribution than the latter. The amino acid residues near the active sites had the greatest effect on the binding of sitagliptin―ACE2. Hydrogen bond as an important indicator of binding capacity had been included in this study. The number and duration of hydrogen bonds in sitagliptin―ACE2 were significantly higher than those in BAR708―ACE2. Therefore, The MD results proved that sitagliptin―ACE2 complex had good stability, and sitagliptin might have a longer binding time than BAR708―ACE2.

### 3.5 SPR assay analysis of sitagliptin binding to ACE2

SPR assay analysis was carried out using Biacore T200 instrument to explore the affinity and kinetic characteristics of sitagliptin binding to ACE2.

The isoelectric point of ACE2 protein was 5.36 calculated by Expasy website. The preconcentration experiments of ACE2 protein were carried out by using different acetate buffer. When pH = 4, the preconcentration platform period of ACE2 protein was reached, and then ACE2 was coupled to the CM5 chip. With 12.5 μM sitagliptin as the initial concentration, three concentrations were diluted downward by twice the gradient, and 0 concentration was set. The solvent correction curve was added to eliminate the influence of solvent on the experiment. The Biacore T200 Control Software was run, the affinity and kinetics of ACE2―sitagliptin were tested by LMW kinetics module. Running Biacore T200 Evaluation Software, the Affinity module was used to analyze the result file to determine whether there was affinity and specific binding between ACE2 and sitagliptin, and obtained the date of affinity and kinetic characteristics.

**Figure 5:**
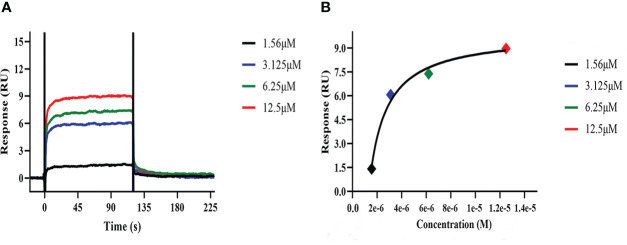
The affinity and kinetic analysis of ACE2―sitagliptin. **(A)** Response value of interaction between sitagliptin and ACE2 on CM5 chip; **(B)** Affinity fitting curves corresponding to reporting points of gradient concentrations.

It was generally believed that the KD values of protein-small molecule affinity fell within the concentration range of 10^−3^ ~10^−9^ M, indicating that protein can bound to small molecules. KD value was around 10^−3^ M, indicating that the binding of protein to small molecules was weak; KD value was about 10^−6^ M, indicating that the protein had moderate binding with small molecules; KD value was about 10^−9^ M, indicating that the protein had strong binding with small molecules. The KD value of sitagliptin binding to ACE2 was 3.514×10^−8^ M, indicating that they had a strong binding capacity. **Figure 5A** showed that they belonged to the “fast binding/fast dissociation” kinetic characteristics. The guiding significance of clinical administration: low dose can reach saturation, but repeated administration was needed. **Figure 5B** showed that they were specific binding and trended to the platform period after about 6×10^−6^ M. Thus, we verified that sitagliptin can bind ACE2 with strong specificity at the molecular level.

### 3.6 Enrichment analysis of GO function and KEGG pathway

**Figure 6:**
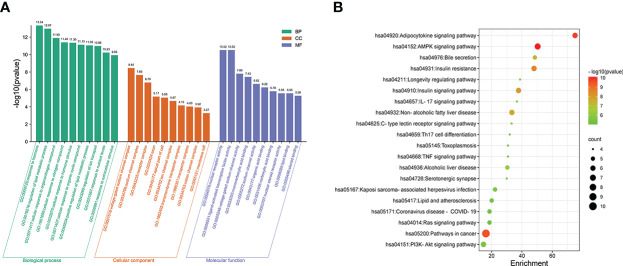
Enrichment analysis of GO function and KEGG pathway. **(A)** GO analysis, including BP, CC, MF; the larger the value of -log10 (pvalue), the more important the item is. **(B)** The larger the value of -log10 (pvalue), the more important the signaling pathway is.

Here, we predicted the molecular mechanism of sitagliptin in the treatment of T2DM. 35 potential targets were imported into Metascape online platform to realize enrichment analysis of GO function and KEGG pathway. Finally, 300 biological processes (BP), 18 cellular components (CC), 44 molecular functions (MF), and 76 KEGG pathways were obtained. The P values of GO functional items were sorted from small to large (**Table S1**), and the top 10 items were visualized by bioinformatics (**Figure 6A**). The results showed that sitagliptin may treat T2DM through the following BP: response to hormone, regulation of lipid metabolic process, cellular response to organonitrogen compound, cellular response to nitrogen compound, cellular response to hormone stimulus, cellular response to organic cyclic compound, positive regulation of lipid metabolic process, regulation of ion transport, response to nutrient levels, response to extracellular stimulus. Sitagliptin in the treatment of T2DM may be associated with the following CC: voltage-gated sodium channel complex, sodium channel complex, receptor complex, axon, apical part of cell, ion channel complex, transmembrane transporter complex, transporter complex, cation channel complex, membrane raft. Sitagliptin in the treatment of T2DM may be associated with the following MF: nuclear receptor activity, ligand-activated transcription factor activity, voltage-gated sodium channel activity, monocarboxylic acid binding, sodium channel activity, organic acid binding, carboxylic acid binding, nuclear steroid receptor activity, lipid binding, steroid binding.

In addition, the P values of KEGG items were sorted from small to large (**Table S2**), and the top 20 items were visualized by bioinformatics (**Figure 6B**). The results showed that sitagliptin may treat T2DM through the following signaling pathway: AMPK signaling pathway, adipocytokine signaling pathway, pathways in cancer, insulin resistance, insulin signaling pathway, non-alcoholic fatty liver disease, bile secretion, alcoholic liver disease, kaposi sarcoma-associated herpesvirus infection, PI3K-Akt signaling pathway, longevity regulating pathway, IL-17 signaling pathway, lipid and atherosclerosis, C-type lectin receptor signaling pathway, ras signaling pathway, coronavirus disease―COVID-19, Th17 cell differentiation, TNF signaling pathway, toxoplasmosis, serotonergic synapse.

*Note:* (A) GO analysis, including BP, CC, MF; the larger the value of -log10 (pvalue), the more important the item is. (B) The larger the value of -log10 (pvalue), the more important the signaling pathway is.

### 3.7 Construction of drug―target―signaling pathway―disease network

**Figure 7:**
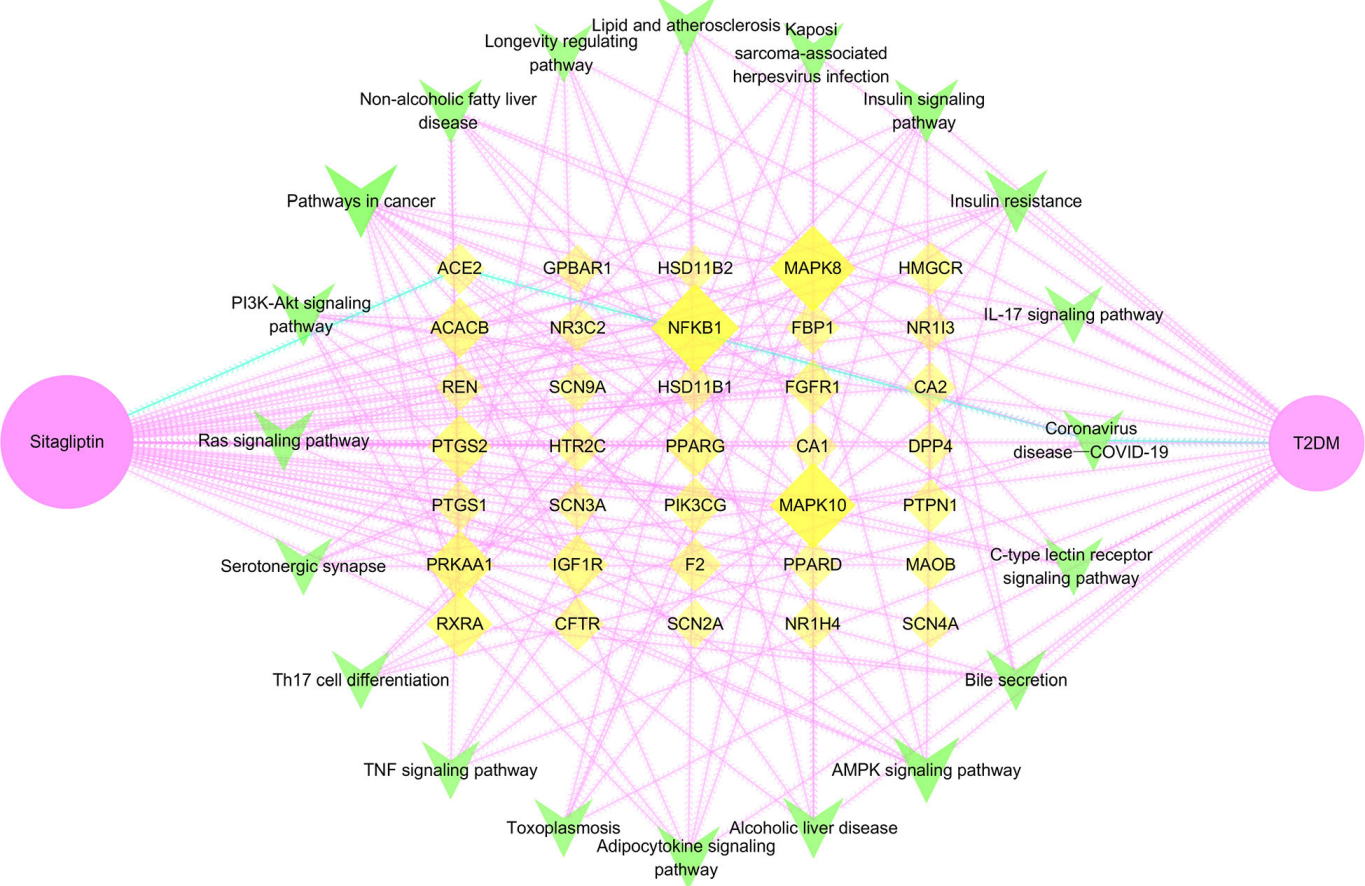
Drug―target―signaling pathway―disease network. Pink node is sitagliptin, yellow node is target, green node is signaling pathway, blue edge is the pathway of sitagliptin targeting ACE2 for T2DM; larger and darker areas indicate more important nodes.

Sitagliptin, 35 potential targets, 20 key signaling pathways and T2DM were imported into Cytoscape software to show the relationship between drug―target―signaling pathway―disease in the form of network topology (**Figure 7**), and further to study the molecular mechanism of sitagliptin in the treatment of T2DM, and to explore the role of ACE2.

Through network topology analysis (**Table S3**), it was found that the network contained 57 nodes and 320 edges. In addition, the average degree of target was 4, and 9 targets were higher than this value: PPARG, IGF1R, RXRA, ACACB, NFKB1, PTGS2, MAPK8, MAPK10, PRKAA1. The average degree of signaling pathway was 6.25, and 7 signaling pathways were higher than this value: AMPK signaling pathway, adipocytokine signaling pathway, pathways in cancer, insulin resistance, insulin signaling pathway, non-alcoholic fatty liver disease, PI3K-Akt signaling pathway. The network reflected the correlation and integrity of multi-targets and multi-signaling pathways in drug treatment of diseases. However, coronavirus disease―COVID-19, the only signaling pathway closely related to ACE2 was included in the follow-up study. Furthermore, we analyzed the coronavirus disease―COVID-19 signaling pathway through the KEGG online platform and speculated that the hypoglycemic effect of sitagliptin may be closely related to the ACE2/Ang-(1-7)/Mas receptor (MasR) axis (**Figure S1**).

*Note:* Pink node is sitagliptin, yellow node is target, green node is signaling pathway, blue edge is the pathway of sitagliptin targeting ACE2 for T2DM; larger and darker areas indicate more important nodes.

## 4 Discussion

Diabetes and its complications bring great suffering to patients around the world, seriously affecting people’s daily lives. Accordingly, diabetes related basic research is particularly important. This study explored new mechanisms and found new targets from old drugs, and provided new reference for the development of targeted drugs.

Through PPI network, we found that ACE2 had the greatest correlation with DPP4 from 35 potential therapeutic targets, so it was used as the research object for further research. Also, ACE2 and DPP4 were both cell surface receptor proteins, which increased the possibility of our speculation. Then, we used semi-flexible molecular docking technology to realize the virtual docking of sitagliptin―ACE2 and BAR708―ACE2. The results showed that sitagliptin could well combine with ACE2, and could be well embedded in the active pocket. The main forces were van der Waals, conventional hydrogen bond, carbon hydrogen bond, halogen (fluorine), alkyl, pi-alkyl and pi-sigma. Furthermore, we used molecular dynamics simulation technology to further verify it. Compared with BAR708―ACE2, sitagliptin―ACE2 had better stability, more hydrogen bonds and longer survival time of hydrogen bonds, which was consistent with the results of molecular docking. It can be seen from **Figure 4** that the changes of energy and RMSF value of the two complex systems were generally consistent, indicating that the biological effects of sitagliptin and BAR708 on ACE2 might be similar in the simulated real aqueous solution environment. In addition, in the free binding energy decomposition of the last frame conformation, it was found that the results in BAR708―ACE2 were unsatisfactory, but the results in sitagliptin―ACE2 performed better. ASP206 and GLU208 were beneficial to the binding of sitagliptin, while ARG219 and LYS562 showed that they were not conducive to the binding of sitagliptin―ACE2. Interestingly, in the BAR708-ACE2 complex, Fiorillo also found that BAR708 bound to the backbone of GLU208 in a hydrogen bond manner, which played an important role in the stability and activity of the complex (22). We also compared three different states of ACE2: natural opening (PDB ID: 1R42), inhibitor closure (PDB ID: 1R4L), and agonist opening (**Figure S2**). From the conformation of the last frame, there was no motion of subdomain I toward subdomain II in the presence of sitagliptin, and ACE2 still showed an open state.

In addition, we explored the affinity and kinetic characteristics of sitagliptin―ACE2 by SPR assay analysis. The high-precision molecular interaction instrument (Biacore T200) provided reliable data support for this study. **Figure 5** showed that there was a strong affinity between sitagliptin and ACE2, the response value of affinity was dose-dependent, and the kinetic characteristic was “fast binding/fast dissociation”. It was suggested that low-dose sitagliptin (about 6×10^−6^ M) can achieve saturated efficacy and dissociate quickly, which may require repeated administrations.

Enrichment analysis of GO function and KEGG pathway were performed on 35 potential targets, and a drug―target―signaling pathway―disease network was constructed to find the action pathway of sitagliptin targeting ACE2 for T2DM: ACE2/Ang-(1-7)/MasR axis in coronavirus disease―COVID-19 signaling pathway. Early studies have observed that ACE2 was mainly localized in the heart, kidney and testis, and was expressed at a low level in many other tissues, especially in the colon and lung (29). Later studies have also shown that ACE2 also played an important role in liver, intestine and other organs. ACE2 was an enzyme on the cell surface, and it was proved to be a key receptor for SASRS-CoV-2 invading the host cells (30). When SARS-CoV-2 invaded the host cells through ACE2, the biological activity of ACE2 in the renin-angiotensin system (RAS) was immediately lost, breaking the steady state of RAS and causing cardiovascular disease in patients with COVID-19 (31, 32). The S protein of coronavirus was a highly glycosylated glycoprotein with a trimer structure, which had three receptor-binding domains (RBD) in a closed state. In the open state, there was only one “upward” conformation of RBD, and the open state was the premise of SARS-CoV-2 fusion with the host cell membrane (33). SARS-CoV-2 recognized and bound to the ACE2 through the RBD of its S1 subunit (34).

Also, some researchers found that the mortality of pulmonary infection in diabetic patients was significantly higher than that in non-diabetic patients, so they believed that there was a correlation between the severity of diabetes and COVID-19 (35, 36). In diabetic state, hyperglycemia activates ACE in the myocardium, generated a large amount of angiotensin II (Ang II), which stimulated the proliferation of myocardial fibroblasts and collagen synthesis, eventually led to myocardial interstitial fibrosis. ACE2 was an ACE homologue discovered in recent years, which can hydrolyze Ang II to Ang- (1–7) (37). ACE played a role in vasoconstriction and fibrosis by binding to AT1, resulting in a significant reduction in insulin secretion, inducing and aggravating diabetes. In contrast to its role was the ACE2/Ang-(1-7)/MasR axis, ACE2 catalyzed Ang II to generate Ang-(1-7), which bound to the MasR, increasing the release of NO by bradykinin. NO played a role in vasodilation, increased islet blood perfusion, thereby improving the blood supply of islets (38). MasR deficiency can lead to decreased expression of glucose transporter 4, decreased insulin sensitivity, decreased utilization of glucose in adipose tissue, and decreased tolerance to high glucose, thereby leading to glucose and lipid metabolism disorders and aggravating insulin resistance (39). Studies have shown that ACE2/Ang-(1-7)/MasR axis can significantly improve liver insulin resistance (40). Ad-hACE2-eGFP significantly improved fasting blood glucose, enhanced intraperitoneal glucose tolerance, increased insulin content and islet β cell proliferation, and reduced islet β cell apoptosis in 8-week-old *db/db* mice through Ang-(1-7)–mediated pathways (41). In addition, the increased expression of ACE2 and Ang-(1-7) can down-regulate the related inflammatory pathways through ACE2/Ang-(1-7)/MasR axis, play a role in anti-inflammatory and anti-fibrosis, and delay the process of diabetic nephropathy (42, 43). Also, the activation of ACE2/Ang-(1-7)/MasR axis can promote the release of NO and improve oxidative stress to protect the renal vascular injury caused by diabetes (44). The results of the clinical trial showed that sitagliptin exerted pharmacological effects such as promoting insulin secretion (6), inhibiting islet β cell apoptosis (45), improving insulin resistance (46), anti-inflammatory (47), anti-oxidative stress (48), and anti-fibrosis (49). It is well known that sitagliptin can specifically inhibit the activity of DPP4 to promote insulin secretion, inhibit islet β cell apoptosis and reduce blood glucose levels, while other pharmacological mechanisms are still unclear (6, 45). In this study, we found that there was a co-expression relationship between ACE2 and DPP4, with a similar protein expression profile (**Figure 2**). Sitagliptin may specifically stimulate the activity of ACE2 through ACE2/Ang-(1-7)/MasR axis to promote insulin secretion, inhibit islet β cell apoptosis and reduce blood glucose levels, which may be synergistic with DPP4-related pharmacological activity. Also, sitagliptin may improve insulin resistance, inflammation, oxidative stress, and fibrosis through ACE2/Ang-(1-7)/MasR axis to treat T2DM and its complications.

In conclusion, we confirmed that sitagliptin can bind to ACE2 and they belonged to “fast binding/fast dissociation” kinetic characteristics. ACE2/Ang-(1-7)/MasR axis was predicted to be the important signaling pathway of sitagliptin targeting ACE2 for T2DM. This study extended the application of ACE2 as a drug target in the treatment of T2DM, based on which the development of drugs or dosage forms was a promising direction.

## Data availability statement

The original contributions presented in the study are included in the article/**Supplementary Material**. Further inquiries can be directed to the corresponding author.

## Author contributions

J-HQ: Conceptualization, Methodology, Software, Investigation, Writing - Original Draft. P-YC and D-YC: Methodology, Investigation, Writing - Original Draft. YW, Y-LW, S-PH: Investigation, Validation, Formal analysis. WZ: Conceptualization, Methodology, Supervision, Writing - Review & Editing. All authors contributed to the article and approved the submitted version.

## Supplementary material

Supplementary Figure 1 ACE2/Ang-(1-7)/MasR axis in coronavirus disease―COVID-19 signaling pathway *Note:* Sitagliptin may specifically stimulate the activity of ACE2 through ACE2/Ang-(1-7)/MasR axis to treat T2DM and its complications. Some pharmacological mechanisms of sitagliptin in clinical trials may be showed through ACE2: promoting insulin secretion, inhibiting islet β cell apoptosis, reducing blood glucose levels, improving insulin resistance, anti-inflammatory, anti-oxidative stress and anti-fibrosis.

Supplementary Figure 2 Three different states of ACE2 *Note:* Natural opening and agonist opening are the active states of ACE2, and inhibitor closure is the inactive state of ACE2.
