# Supplementary figures and images for: Exploring novel targets of sitagliptin for type 2 diabetes mellitus: Network pharmacology, molecular docking, molecular dynamics simulation, and SPR approaches
Source: Front Endocrinol (Lausanne). 2023 Jan 9;13:1096655. doi: 10.3389/fendo.2022.1096655 (PMC9868454; doi:10.3389/fendo.2022.1096655)

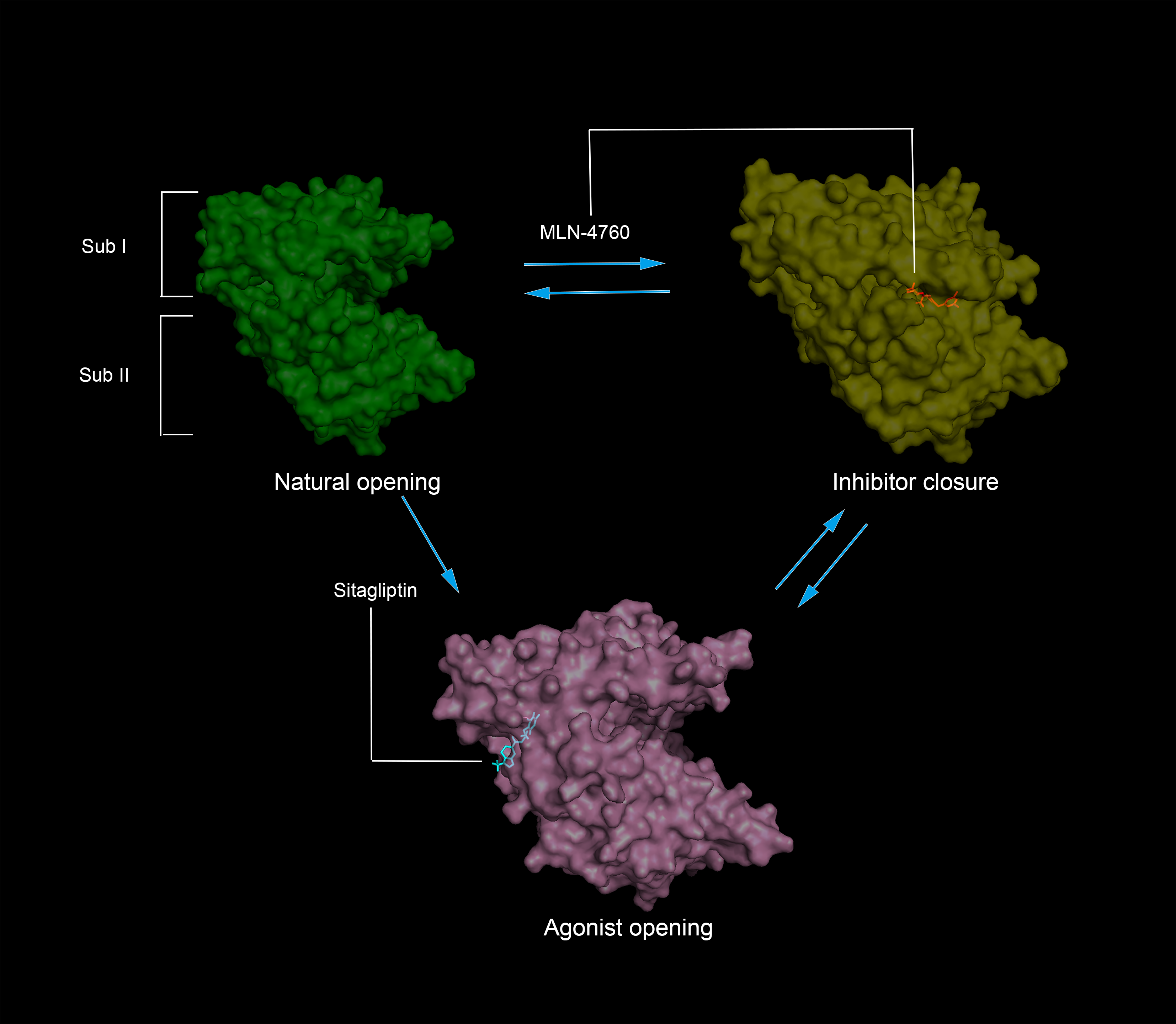

Supplement: Supplementary file 1 [file Image_3.tif]

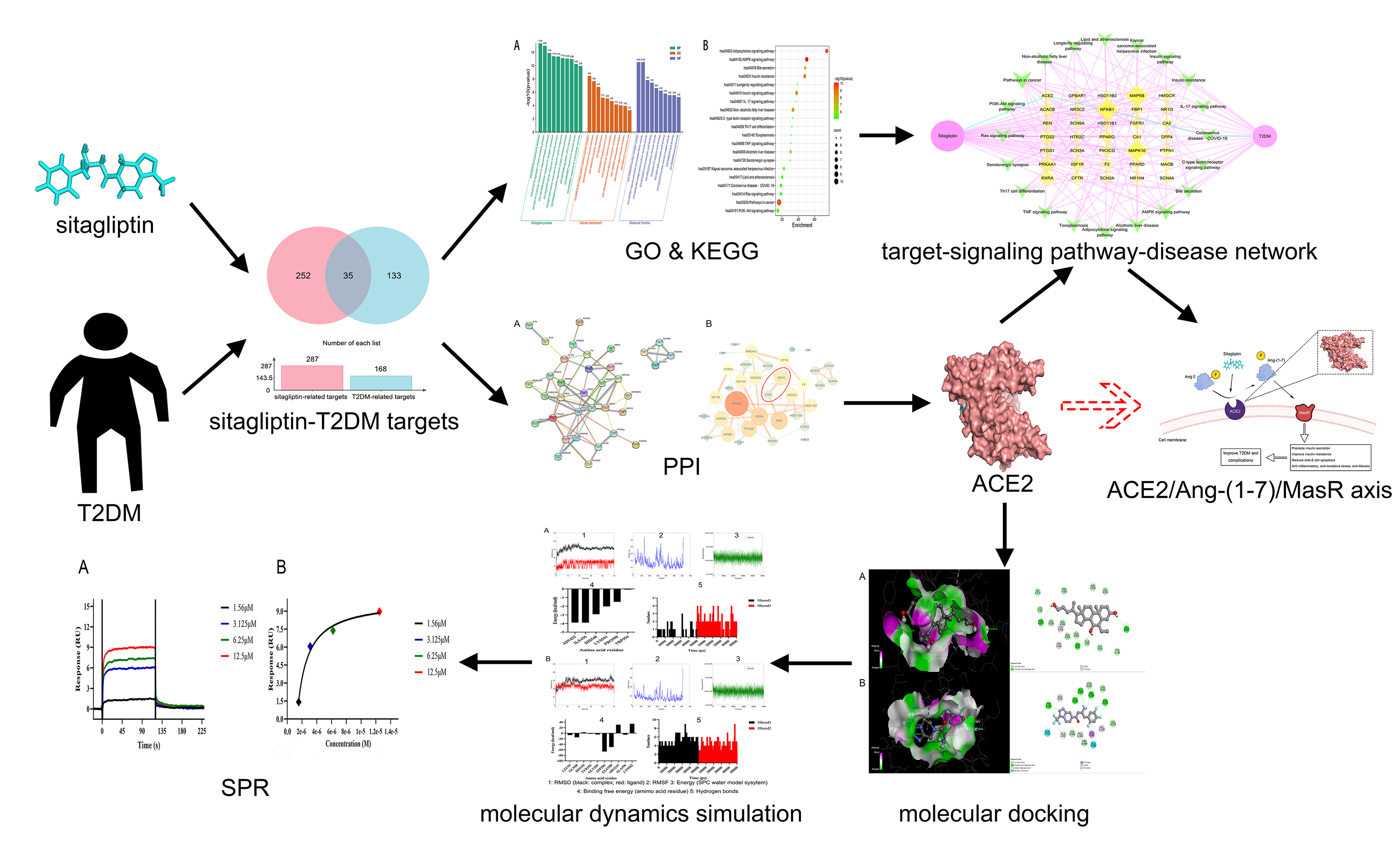

Supplement: Supplementary Figure 1 — ACE2/Ang-(1-7)/MasR axis in coronavirus disease―COVID-19 signaling pathway Note: Sitagliptin may specifically stimulate the activity of ACE2 through ACE2/Ang-(1-7)/MasR axis to treat T2DM and its complications. Some pharmacological mechanisms of sitagliptin in clinical trials may be showed through ACE2: promoting insulin secretion, inhibiting islet β cell apoptosis, reducing blood glucose levels, improving insulin resistance, anti-inflammatory, anti-oxidative stress and anti-fibrosis. [file Image_1.tif]

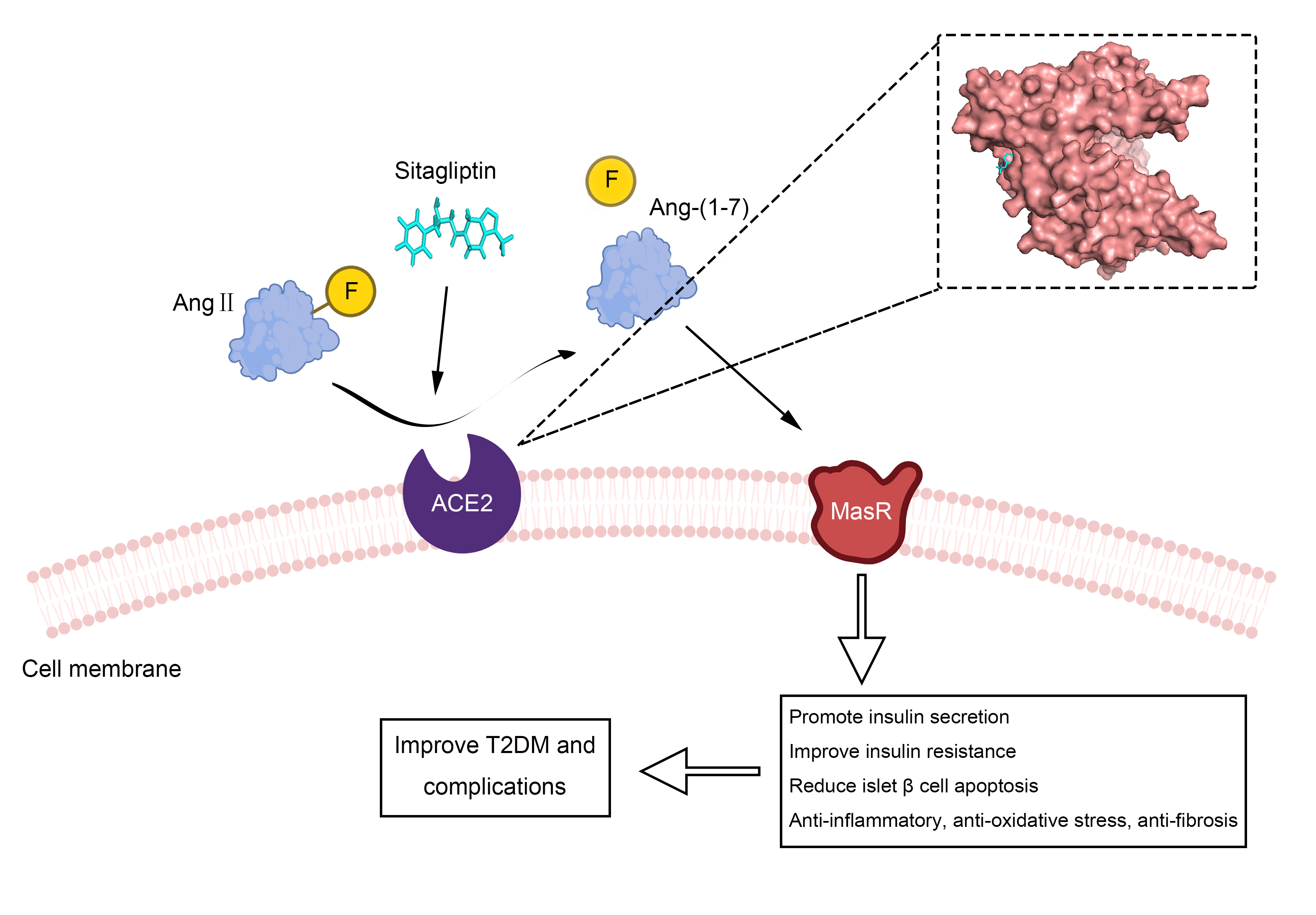

Supplement: Supplementary Figure 2 — Three different states of ACE2 Note: Natural opening and agonist opening are the active states of ACE2, and inhibitor closure is the inactive state of ACE2. [file Image_2.tif]
