# Supplementary material for: Exploring novel targets of sitagliptin for type 2 diabetes mellitus: Network pharmacology, molecular docking, molecular dynamics simulation, and SPR approaches
Source: Front Endocrinol (Lausanne). 2023 Jan 9;13:1096655. doi: 10.3389/fendo.2022.1096655 (PMC9868454; doi:10.3389/fendo.2022.1096655)
Supplement: Supplementary file 2 [file Table_1.docx]

**Table S1.** Annotation information of GO function

| **Term** | **Description** | **subgroup** | **LogP** | **Log(q-value)** | **Symbols** |
| --- | --- | --- | --- | --- | --- |
| GO:0009725 | response to hormone | BP | −13.34123909 | −8.988265912 | CA2,FBP1,HSD11B2,IGF1R,MAOB,NR3C2,NFKB1,PPARD,PPARG,PRKAA1,PTGS2,PTPN1,REN,RXRA |
| GO:0019216 | regulation of lipid metabolic process | BP | −12.97400251 | −8.922059324 | ACACB,F2,HTR2C,IGF1R,NFKB1,PIK3CG,PPARD,PPARG,PRKAA1,PTGS2,NR1H4 |
| GO:0071417 | cellular response to organonitrogen compound | BP | −11.90087502 | −8.025023101 | CA2,CFTR,FBP1,HTR2C,IGF1R,NFKB1,PIK3CG,PPARG,PRKAA1,PTGS2,PTPN1,NR1H4 |
| GO:1901699 | cellular response to nitrogen compound | BP | −11.40178637 | −7.700729386 | CA2,CFTR,FBP1,HTR2C,IGF1R,NFKB1,PIK3CG,PPARG,PRKAA1,PTGS2,PTPN1,NR1H4 |
| GO:0032870 | cellular response to hormone stimulus | BP | −11.35473256 | −7.700729386 | CA2,FBP1,IGF1R,NR3C2,NFKB1,PPARD,PPARG,PRKAA1,PTPN1,REN,RXRA |
| GO:0071407 | cellular response to organic cyclic compound | BP | −11.12742181 | −7.552599881 | CFTR,HTR2C,IGF1R,NR3C2,NFKB1,PIK3CG,PPARD,PTGS2,RXRA,NR1H4,GPBAR1 |
| GO:0045834 | positive regulation of lipid metabolic process | BP | −11.05311852 | −7.545243379 | F2,HTR2C,IGF1R,PPARD,PPARG,PRKAA1,PTGS2,NR1H4 |
| GO:0043269 | regulation of ion transport | BP | −10.97840214 | −7.52851895 | CA2,CFTR,F2,MAOB,PIK3CG,PRKAA1,PTGS2,SCN2A,SCN3A,SCN4A,SCN9A,ACE2 |
| GO:0031667 | response to nutrient levels | BP | −10.22945245 | −6.917871953 | ACACB,HSD11B2,HTR2C,IGF1R,PPARD,PPARG,PRKAA1,MAPK8,PTGS2,NR1H4 |
| GO:0009991 | response to extracellular stimulus | BP | −9.927296491 | −6.688266664 | ACACB,HSD11B2,HTR2C,IGF1R,PPARD,PPARG,PRKAA1,MAPK8,PTGS2,NR1H4 |
| GO:0001518 | voltage-gated sodium channel complex | CC | −8.448677543 | −5.380783926 | SCN2A,SCN3A,SCN4A,SCN9A |
| GO:0034706 | sodium channel complex | CC | −7.65382465 | −4.806001449 | SCN2A,SCN3A,SCN4A,SCN9A |
| GO:0043235 | receptor complex | CC | −6.791459859 | −4.186674707 | FGFR1,HTR2C,IGF1R,NR3C2,PPARG,RXRA,NR1H4,GPBAR1 |
| GO:0030424 | axon | CC | −5.167290122 | −2.908857062 | IGF1R,PRKAA1,MAPK8,SCN2A,SCN3A,SCN4A,SCN9A |
| GO:0045177 | apical part of cell | CC | −5.047225196 | −2.821532531 | CA2,CFTR,DPP4,PRKAA1,REN,ACE2 |
| GO:0034702 | ion channel complex | CC | −4.665496012 | −2.522037848 | CFTR,SCN2A,SCN3A,SCN4A,SCN9A |
| GO:1902495 | transmembrane transporter complex | CC | −4.152139538 | −2.113033579 | CFTR,SCN2A,SCN3A,SCN4A,SCN9A |
| GO:1990351 | transporter complex | CC | −4.031164232 | −2.0361259 | CFTR,SCN2A,SCN3A,SCN4A,SCN9A |
| GO:0034703 | cation channel complex | CC | −3.920064017 | −1.943667795 | SCN2A,SCN3A,SCN4A,SCN9A |
| GO:0045121 | membrane raft | CC | −3.270744531 | −1.412150012 | DPP4,IGF1R,PTGS2,ACE2 |
| GO:0004879 | nuclear receptor activity | MF | −10.51967201 | −7.166698827 | NR3C2,PPARD,PPARG,RXRA,NR1I3,NR1H4 |
| GO:0098531 | ligand-activated transcription factor activity | MF | −10.51967201 | −7.166698827 | NR3C2,PPARD,PPARG,RXRA,NR1I3,NR1H4 |
| GO:0005248 | voltage-gated sodium channel activity | MF | −7.801382416 | −4.925530492 | SCN2A,SCN3A,SCN4A,SCN9A |
| GO:0033293 | monocarboxylic acid binding | MF | −7.427913828 | −4.675879546 | ACACB,PPARD,PPARG,RXRA,NR1H4 |
| GO:0005272 | sodium channel activity | MF | −6.622921209 | −4.040800041 | SCN2A,SCN3A,SCN4A,SCN9A |
| GO:0043177 | organic acid binding | MF | −6.222334514 | −3.761455938 | ACACB,PPARD,PPARG,RXRA,NR1H4 |
| GO:0031406 | carboxylic acid binding | MF | −5.783909189 | −3.413207243 | ACACB,PPARD,PPARG,RXRA,NR1H4 |
| GO:0003707 | nuclear steroid receptor activity | MF | −5.546869966 | −3.21415772 | NR3C2,PPARD,RXRA |
| GO:0008289 | lipid binding | MF | −5.5459416 | −3.21415772 | F2,HSD11B1,HSD11B2,NR3C2,PPARD,PPARG,RXRA,NR1H4 |
| GO:0005496 | steroid binding | MF | −5.261440708 | −2.98401449 | HSD11B1,HSD11B2,NR3C2,NR1H4 |
