# Supplementary material for: Exploring novel targets of sitagliptin for type 2 diabetes mellitus: Network pharmacology, molecular docking, molecular dynamics simulation, and SPR approaches
Source: Front Endocrinol (Lausanne). 2023 Jan 9;13:1096655. doi: 10.3389/fendo.2022.1096655 (PMC9868454; doi:10.3389/fendo.2022.1096655)
Supplement: Supplementary file 3 [file Table_2.docx]

**Table S2.** Annotation information of KEGG pathways

| **Term** | **Description** | **LogP** | **Log(q-value)** | **Symbols** |
| --- | --- | --- | --- | --- |
| hsa04152 | AMPK signaling pathway | −10.0949047 | −6.821112772 | ACACB,CFTR,FBP1,HMGCR,IGF1R,PPARG,PRKAA1 |
| hsa04920 | Adipocytokine signaling pathway | −9.755765432 | −6.578883512 | ACACB,NFKB1,PRKAA1,MAPK8,MAPK10,RXRA |
| hsa05200 | Pathways in cancer | −9.492871238 | −6.344018042 | F2,FGFR1,IGF1R,NFKB1,PPARD,PPARG,MAPK8,MAPK10,PTGS2,RXRA |
| hsa04931 | Insulin resistance | −8.566626003 | −5.468925329 | ACACB,NFKB1,PRKAA1,MAPK8,MAPK10,PTPN1 |
| hsa04910 | Insulin signaling pathway | −7.944063002 | −5.022453587 | ACACB,FBP1,PRKAA1,MAPK8,MAPK10,PTPN1 |
| hsa04932 | Non-alcoholic fatty liver disease | −7.623180012 | −4.80168575 | NFKB1,PPARG,PRKAA1,MAPK8,MAPK10,RXRA |
| hsa04976 | Bile secretion | −7.221270951 | −4.558493852 | CA2,CFTR,HMGCR,RXRA,NR1H4 |
| hsa04936 | Alcoholic liver disease | −6.207126696 | −3.751780608 | ACACB,NFKB1,PRKAA1,MAPK8,MAPK10 |
| hsa05167 | Kaposi sarcoma-associated herpesvirus infection | −5.539928567 | −3.212261253 | NFKB1,PIK3CG,MAPK8,MAPK10,PTGS2 |
| hsa04151 | PI3K-Akt signaling pathway | −5.518497432 | −3.194908031 | FGFR1,IGF1R,NFKB1,PIK3CG,PRKAA1,RXRA |
| hsa04211 | Longevity regulating pathway | −5.463260408 | −3.147713727 | IGF1R,NFKB1,PPARG,PRKAA1 |
| hsa04657 | IL-17 signaling pathway | −5.368493955 | −3.079978765 | NFKB1,MAPK8,MAPK10,PTGS2 |
| hsa05417 | Lipid and atherosclerosis | −5.322086546 | −3.037299228 | NFKB1,PPARG,MAPK8,MAPK10,RXRA |
| hsa04625 | C-type lectin receptor signaling pathway | −5.193708039 | −2.930639972 | NFKB1,MAPK8,MAPK10,PTGS2 |
| hsa04014 | Ras signaling pathway | −5.161459696 | −2.908857062 | FGFR1,IGF1R,NFKB1,MAPK8,MAPK10 |
| hsa05171 | Coronavirus disease - COVID-19 | −5.161459696 | −2.908857062 | F2,NFKB1,MAPK8,MAPK10,ACE2 |
| hsa04659 | Th17 cell differentiation | −5.128623844 | −2.879454386 | NFKB1,MAPK8,MAPK10,RXRA |
| hsa04668 | TNF signaling pathway | −5.065992707 | −2.833593459 | NFKB1,MAPK8,MAPK10,PTGS2 |
| hsa05145 | Toxoplasmosis | −5.065992707 | −2.833593459 | NFKB1,PIK3CG,MAPK8,MAPK10 |
| hsa04726 | Serotonergic synapse | −5.020523467 | −2.804270855 | HTR2C,MAOB,PTGS1,PTGS2 |
