# Supplementary material for: Exploring novel targets of sitagliptin for type 2 diabetes mellitus: Network pharmacology, molecular docking, molecular dynamics simulation, and SPR approaches
Source: Front Endocrinol (Lausanne). 2023 Jan 9;13:1096655. doi: 10.3389/fendo.2022.1096655 (PMC9868454; doi:10.3389/fendo.2022.1096655)
Supplement: Supplementary file 4 [file Table_3.docx]

**Table S3.** Topology information of drug―target―signaling pathway―disease network

| **SUID** | **Name** | **Type** | **Degree** | **AverageShortestPathLength** | **BetweennessCentrality** | **ClosenessCentrality** |
| --- | --- | --- | --- | --- | --- | --- |
| 72 | Sitagliptin | drug | 35 | 1.39285714 | 0.6221778 | 0.71794872 |
| 268 | T2DM | drug | 20 | 2.08928571 | 0.09944986 | 0.47863248 |
| 113 | NFKB1 | gene | 17 | 1.76785714 | 0.0844627 | 0.56565657 |
| 129 | MAPK8 | gene | 16 | 1.80357143 | 0.07443134 | 0.55445545 |
| 131 | MAPK10 | gene | 16 | 1.80357143 | 0.07443134 | 0.55445545 |
| 158 | Pathways in cancer | pathway | 11 | 2.25 | 0.03342568 | 0.44444444 |
| 133 | PRKAA1 | gene | 9 | 2.05357143 | 0.03088804 | 0.48695652 |
| 93 | RXRA | gene | 8 | 2.08928571 | 0.02727841 | 0.47863248 |
| 143 | AMPK signaling pathway | pathway | 8 | 2.35714286 | 0.01872521 | 0.42424242 |
| 121 | PTGS2 | gene | 7 | 2.125 | 0.02532404 | 0.47058824 |
| 151 | Adipocytokine signaling pathway | pathway | 7 | 2.39285714 | 0.00735283 | 0.41791045 |
| 169 | Insulin resistance | pathway | 7 | 2.39285714 | 0.01003686 | 0.41791045 |
| 176 | Insulin signaling pathway | pathway | 7 | 2.39285714 | 0.01305172 | 0.41791045 |
| 183 | Non-alcoholic fatty liver disease | pathway | 7 | 2.39285714 | 0.00727373 | 0.41791045 |
| 208 | PI3K-Akt signaling pathway | pathway | 7 | 2.39285714 | 0.01141101 | 0.41791045 |
| 83 | PPARG | gene | 6 | 2.16071429 | 0.01655617 | 0.46280992 |
| 87 | IGF1R | gene | 6 | 2.16071429 | 0.01701292 | 0.46280992 |
| 111 | ACACB | gene | 6 | 2.16071429 | 0.01553094 | 0.46280992 |
| 190 | Bile secretion | pathway | 6 | 2.42857143 | 0.01483317 | 0.41176471 |
| 196 | Alcoholic liver disease | pathway | 6 | 2.42857143 | 0.00480844 | 0.41176471 |
| 202 | Kaposi sarcoma-associated herpesvirus infection | pathway | 6 | 2.42857143 | 0.00650816 | 0.41176471 |
| 225 | Lipid and atherosclerosis | pathway | 6 | 2.42857143 | 0.0050592 | 0.41176471 |
| 236 | Ras signaling pathway | pathway | 6 | 2.42857143 | 0.00693769 | 0.41176471 |
| 242 | Coronavirus disease—COVID-19 | pathway | 6 | 2.42857143 | 0.01151096 | 0.41176471 |
| 215 | Longevity regulating pathway | pathway | 5 | 2.46428571 | 0.00438242 | 0.4057971 |
| 220 | IL-17 signaling pathway | pathway | 5 | 2.46428571 | 0.00262427 | 0.4057971 |
| 231 | C-type lectin receptor signaling pathway | pathway | 5 | 2.46428571 | 0.00262427 | 0.4057971 |
| 248 | Th17 cell differentiation | pathway | 5 | 2.46428571 | 0.00242301 | 0.4057971 |
| 253 | TNF signaling pathway | pathway | 5 | 2.46428571 | 0.00262427 | 0.4057971 |
| 258 | Toxoplasmosis | pathway | 5 | 2.46428571 | 0.00411197 | 0.4057971 |
| 263 | Serotonergic synapse | pathway | 5 | 2.46428571 | 0.01199343 | 0.4057971 |
| 117 | PIK3CG | gene | 4 | 2.23214286 | 0.00994032 | 0.448 |
| 141 | FGFR1 | gene | 4 | 2.23214286 | 0.00776919 | 0.448 |
| 75 | HMGCR | gene | 3 | 2.26785714 | 0.00573272 | 0.44094488 |
| 85 | CFTR | gene | 3 | 2.26785714 | 0.00573272 | 0.44094488 |
| 109 | F2 | gene | 3 | 2.26785714 | 0.00479348 | 0.44094488 |
| 137 | FBP1 | gene | 3 | 2.26785714 | 0.00503985 | 0.44094488 |
| 139 | PTPN1 | gene | 3 | 2.26785714 | 0.00512398 | 0.44094488 |
| 79 | HTR2C | gene | 2 | 2.30357143 | 0.00376696 | 0.43410853 |
| 89 | MAOB | gene | 2 | 2.30357143 | 0.00376696 | 0.43410853 |
| 91 | PPARD | gene | 2 | 2.30357143 | 0.00146797 | 0.43410853 |
| 103 | CA2 | gene | 2 | 2.30357143 | 0.00298335 | 0.43410853 |
| 115 | ACE2 | gene | 2 | 2.30357143 | 0.00293529 | 0.43410853 |
| 119 | NR1H4 | gene | 2 | 2.30357143 | 0.00298335 | 0.43410853 |
| 123 | PTGS1 | gene | 2 | 2.30357143 | 0.00376696 | 0.43410853 |
| 73 | DPP4 | gene | 1 | 2.375 | 0 | 0.42105263 |
| 77 | NR1I3 | gene | 1 | 2.375 | 0 | 0.42105263 |
| 81 | NR3C2 | gene | 1 | 2.375 | 0 | 0.42105263 |
| 95 | SCN2A | gene | 1 | 2.375 | 0 | 0.42105263 |
| 97 | SCN3A | gene | 1 | 2.375 | 0 | 0.42105263 |
| 99 | SCN4A | gene | 1 | 2.375 | 0 | 0.42105263 |
| 101 | SCN9A | gene | 1 | 2.375 | 0 | 0.42105263 |
| 105 | CA1 | gene | 1 | 2.375 | 0 | 0.42105263 |
| 107 | HSD11B1 | gene | 1 | 2.375 | 0 | 0.42105263 |
| 125 | GPBAR1 | gene | 1 | 2.375 | 0 | 0.42105263 |
| 127 | HSD11B2 | gene | 1 | 2.375 | 0 | 0.42105263 |
| 135 | REN | gene | 1 | 2.375 | 0 | 0.42105263 |
